# Supplementary material for: Neutrophil Extracellular Trap Formation Correlates with Favorable Overall Survival in High Grade Ovarian Cancer
Source: Cancers (Basel). 2020 Feb 21;12(2):505. doi: 10.3390/cancers12020505 (PMC7072166; doi:10.3390/cancers12020505)
Supplement: Supplementary file 1 [file cancers-12-00505-s001.zip › Supplementary Figures.docx]

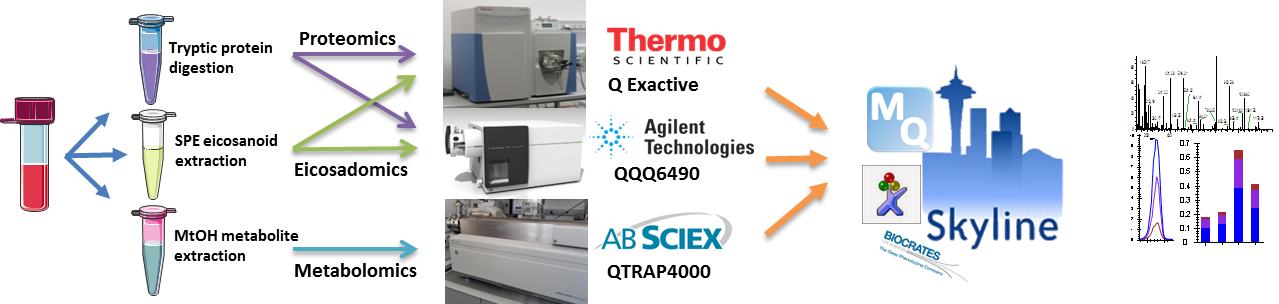


**Figure S1: Workflow of multi-omics analysis.** The multi-omics analysis was performed with ascites samples and supernatant of neutrophils. In each sample proteins, eicosanoids and metabolites were measured. Shotgun proteomics analysis of depleted ascites samples following protein digestion was conducted on a Q Exactive Orbitrap mass spectrometer coupled with a nano-LC system. MaxQuant software tools were used for protein identification and MS1 based protein quantification. After precipitation of proteins with ethanol from ascites samples, eicosanoids were isolated using SPE extraction technique. Eicosadomics screening analysis was performed on a Q Exactive Orbitrap mass spectrometer coupled with a UHPLC system. Eicosanoids were manually identified based on MS/MS spectra using Xcalibur software. However, the correct eicosanoid identification was confirmed with synthetic standards. Skyline software was used for abundance assessment at MS1 level. The Biocrates kit was implemented for the analysis of metabolites. A targeted multi-omics approach conducted on triple quadrupole mass spectrometer was applied for the analysis of supernatant samples isolated from neutrophils. All targeted multi-omics data were evaluated with Skyline software.


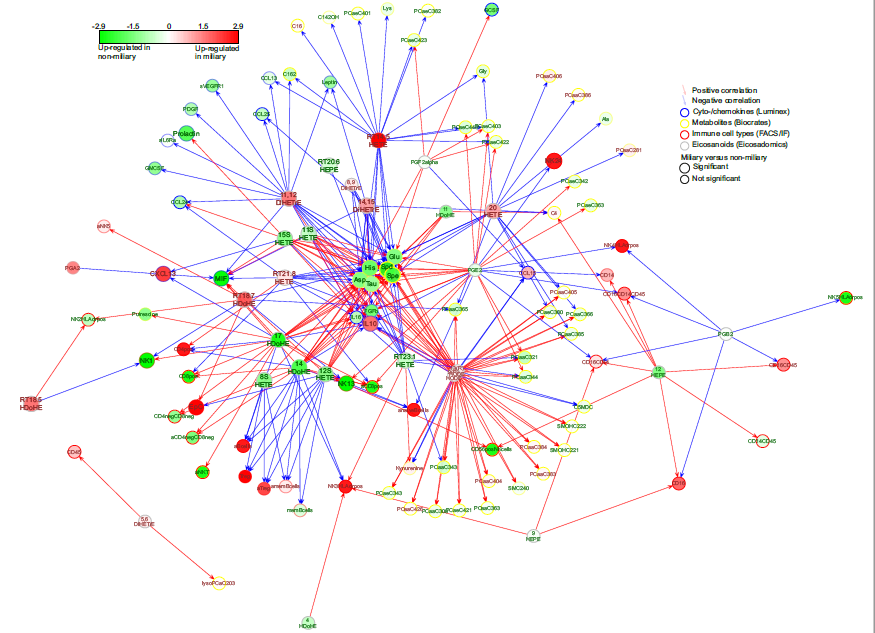


**Figure S2：**Whole network signature


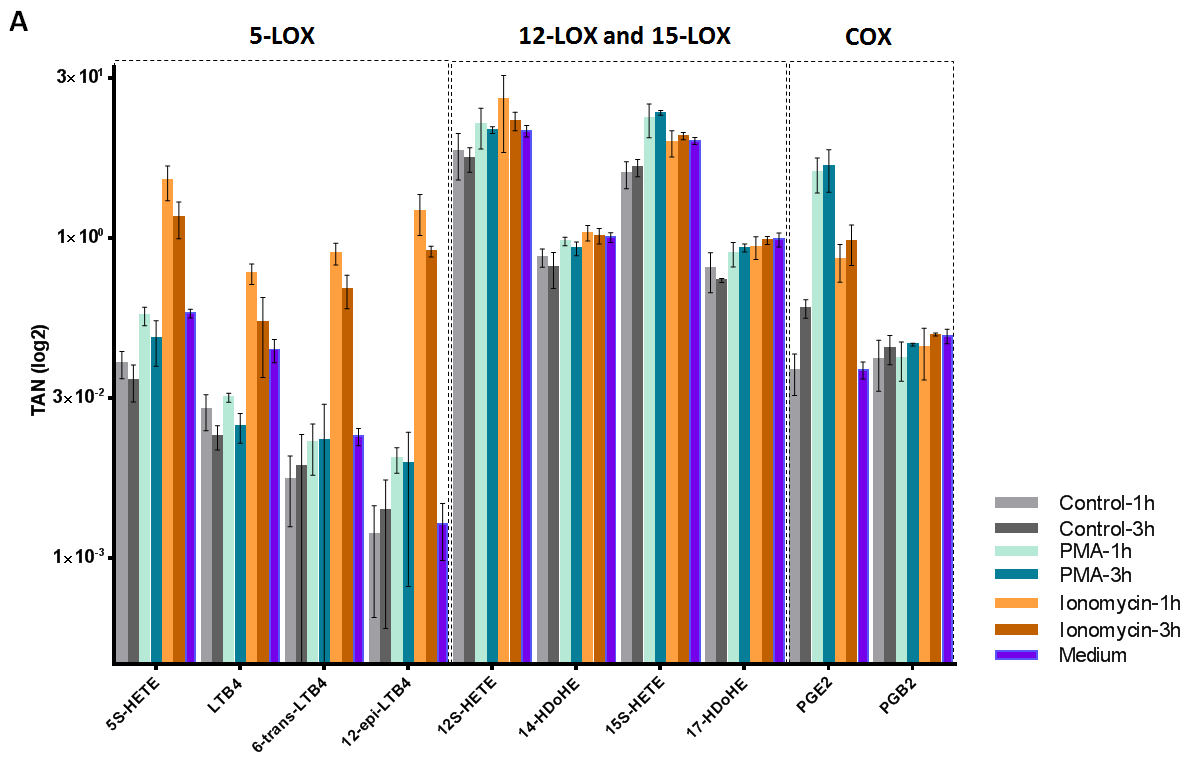


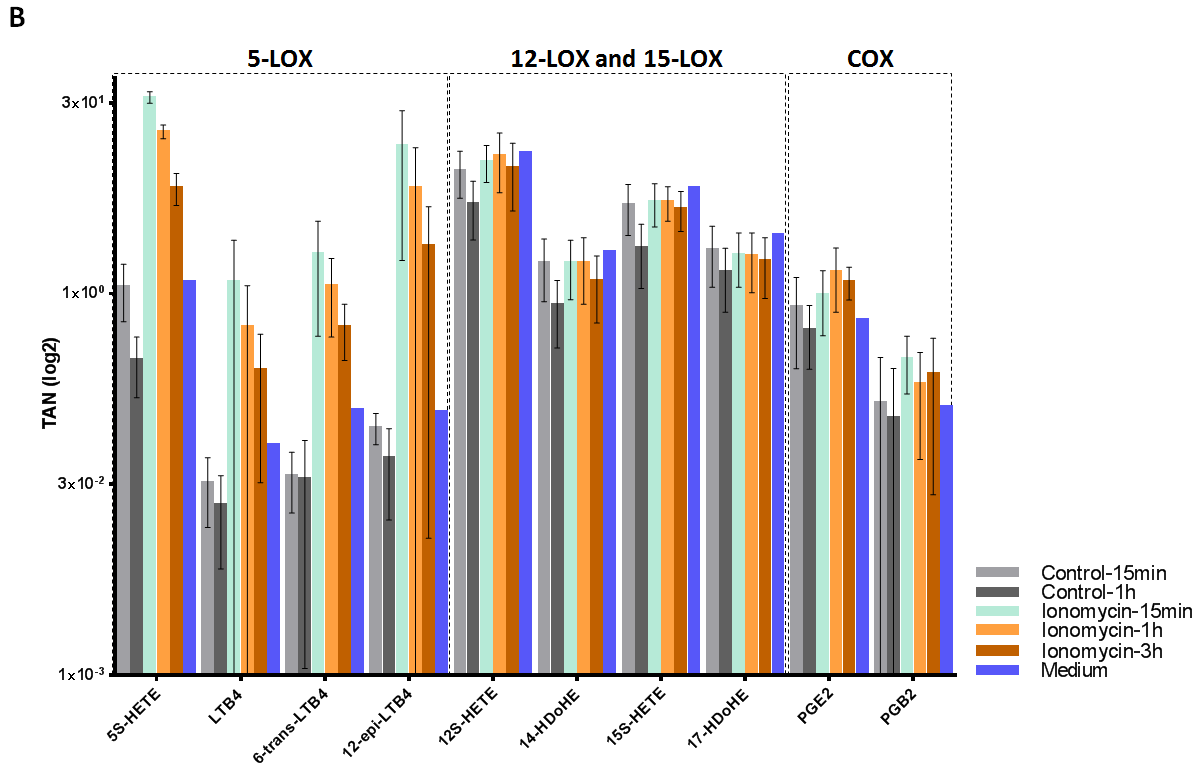


**Figure S3: Results of ecosadomics analysis with supernatant samples of neutrophils isolated from healthy donors. A -** Neutrophils were cultured in a medium supplemented with 10% FCS. Cells isolated from healthy donors (n=3) were treated with PMA (25nM) or ionomycin (4µM) and a targeted MS approach was implemented for the analysis of the supernatant samples. When comparing ionomycin treated samples with their respective untreated control samples, all 5-LOX products and PGE2 were significantly up-regulated. On the other hand, 12-epi-LTB4 and PGE2 were significantly up-regulated upon PMA treatment. A p-value lower than 0.05 was defined as a criterion for significant regulation. **B** **-** Neutrophils (n = 5 ) were treated only with 4µM ionomycin for 15 minutes, 1 hour or 3 hours. The data were generated using a Q Exactive HF mass spectrometer by applying an untargeted approach, and the abundance was assessed based on MS1 spectra. Error bars indicate standard deviation. Among all measured eicosanoids and at least at one of three treatment conditions, only 5-LOX products and PGE2 were significantly up-regulated.


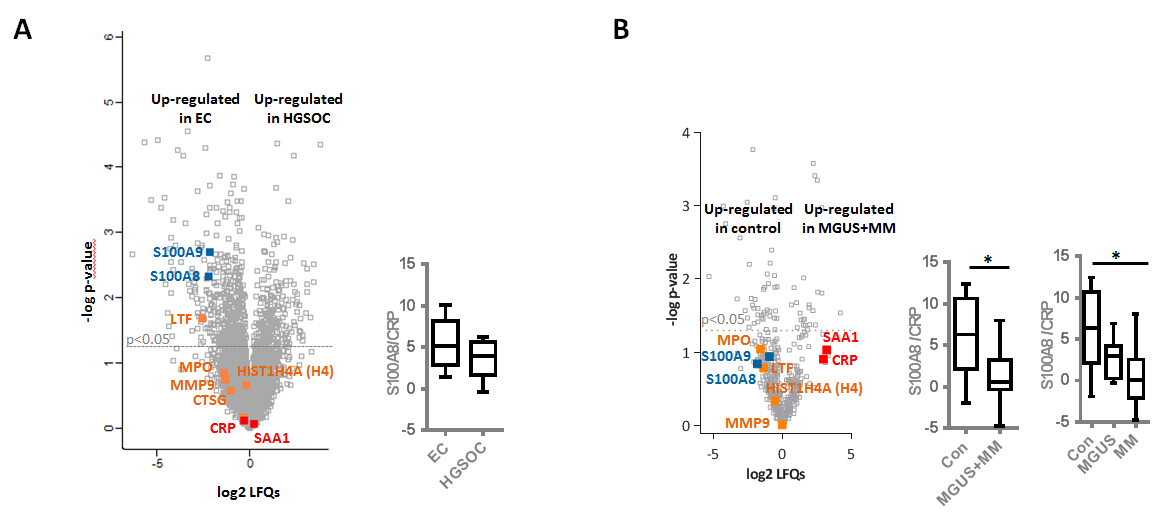


**Figure S4: Shotgun proteomics.**  Data were generated by the analysis of tissue samples taken from ovarian cancer patients **(A)** and bone marrow plasma samples of multiple myeloma patients **(B)**. The volcano plots represent protein differences between compared patients, while boxplots show the distribution of the ratio. A more favorable outcome characterizes endometrioid carcinoma (EC) compared to HGSOC. MGUS is a pre-stage of multiple myeloma (MM). * - indicate p-value < 0.05.


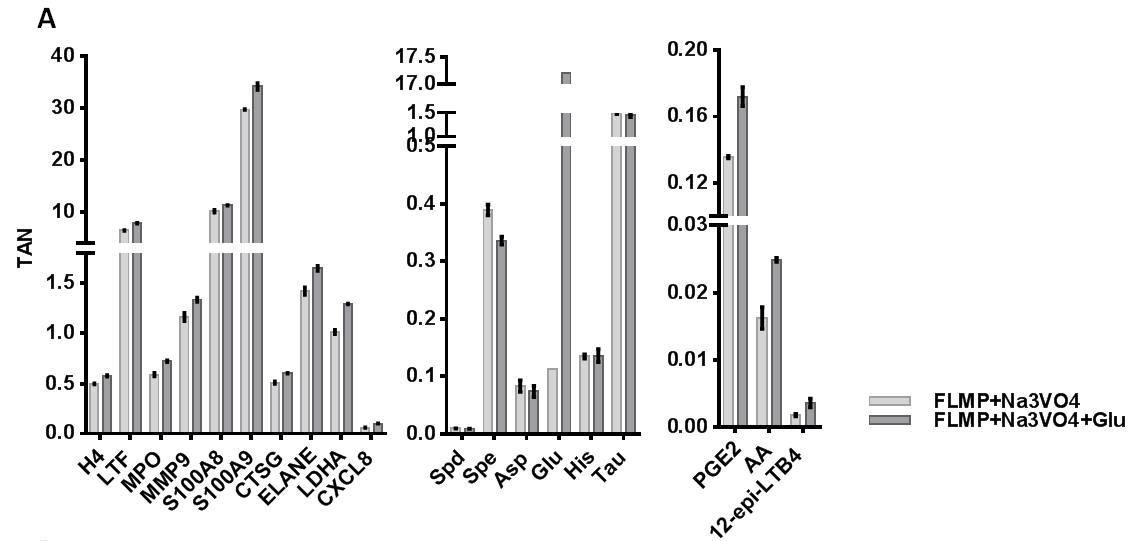


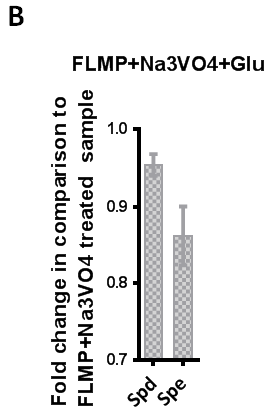


**Figure S5: Targeted multi-omics analysis of neutrophils treated with glutamate.** Neutrophils isolated from a healthy donor were first primed by 15 minutes treatment with 10^-11^ M FLMP. After that, the control cells were treated only with 100µM Na_3_PO_4_ for 3 hours and to the other cells were additionally added Glu to the final concentration of 250µM. The TAN values for both applied treatment conditions of molecules of interest were shown **(A)**. The fold changes for Spe and Spd in Glu treated compared to control samples **(B)**.
